# Effect of Serial Anthropometric Measurements and Motivational Text Messages on Weight Reduction Among Workers: Pilot Randomized Controlled Trial

**DOI:** 10.2196/11832

**Published:** 2019-04-24

**Authors:** Renee Chan, Matthew Nguyen, Rachel Smith, Sarah Spencer, Sabrina Winona Pit

**Affiliations:** 1 School Of Medicine University Centre for Rural Health Western Sydney University Lismore Australia; 2 Rural Clinical School University of Sydney Lismore Australia

**Keywords:** text messages, obesity, waist-hip ratio, weight reduction programs, mHealth

## Abstract

**Background:**

Obesity is an endemic problem with significant health and financial consequences. Text messaging has been shown to be a simple and effective method of facilitating weight reduction. In addition, waist-to-hip ratio (WHR) has emerged as a significant anthropometric measure. However, few studies have examined the effect of serial anthropometric self-measurement combined with text messaging.

**Objective:**

The primary aim of this study was to assess whether an 8-week program, consisting of weekly serial self-measurements of waist and hip circumference, combined with motivational text messages, could reduce WHR among Australian workers.

**Methods:**

This was a community-based, participant-blinded, staggered-entry, parallel group study. Adult workers with access to mobile phones were eligible and recruited through an open access Web-based survey. Participants were randomly allocated to receive intervention or control messages for 8 weeks. Outcome data were self-assessed through a Web-based survey.

**Results:**

A total of 60 participants were randomized with 30 participants each allocated to a control and an intervention group. There was no significant change in WHR (*P*=.43), and all secondary outcome measures did not differ between the intervention group and the control group at the end of the 8-week intervention. Both groups, however, showed a significant decrease in burnout over time (mean [SE]: pre 4.80 [0.39] vs post 3.36 [0.46]; *P*=.004). The intervention uptake followed a downward trend. Peak participant replies to weekly self-measurements were received in week 3 (14/23, 61%) and the least in week 8 (8/23, 35%). No harm was found to result from this study.

**Conclusions:**

This study is an innovative pilot trial using text messaging and serial anthropometric measurements in weight management. No change was detected in WHRs in Australian workers over 8 weeks; therefore, it could not be concluded whether the intervention affected the primary outcome. However, these results should be interpreted in the context of limited sample size and decreasing intervention uptake over the course of the study. This pilot trial is useful for informing and contributing to the design of future studies and the growing body of literature on serial self-measurements combined with text messaging.

**Trial Registration:**

Australian New Zealand Clinical Trials Registry ACTRN12616001496404; https://www.anzctr.org.au/Trial/Registration/TrialReview.aspx?id=371696&isReview=true (Archived by WebCite at http://www.webcitation.org/73UkKFjSw)

## Introduction

### The Problem of Obesity

Obesity is an endemic problem worldwide with significant health consequences to the individual [1] and financial burden on the community. The economic cost of obesity in Australia was estimated at Aus $52.8 billion in 2008 alone, including productivity loss, costs to the health system, and impact on well-being [2]. In 2014-15, 28% of Australian adults were obese, showing an increase of 19% from 1995 [3]. Obesity is commonly measured by weight and body mass index (BMI); however, waist-to-hip ratio (WHR) is also increasingly being recognized as being an important measure of obesity [4].

### Text Messaging in Weight Loss

Exercise is a key element in achieving weight loss goals; however, there are many influences on an individual’s level of exercise, including perceptions of support [5]. Text messaging has proven to be a cheap, simple, and an effective support strategy in encouraging weight reduction [6-8]. Text messages used to promote health messages currently have limited evidence but show promising potential as an effective health promotion tool [9]. In 1 study, tailored text messages and multimedia messaging service with tips, suggestions, and positive reinforcement over 4 months led to an additional loss of 2 kg in the intervention compared with the control group that received monthly printed materials over a period of 4 months [10]. Moreover, there is some evidence that 1 text message a day is able to improve motivation toward weight loss behaviors without adding extra burden, but this requires further testing [9].

There is no consensus with respect to the most effective text message content. Interventions to increase physical activity and healthy eating vary widely. Despite this, there are few systematic reviews evaluating factors that influence the effectiveness of text message interventions. There is a taxonomy of behavioral change techniques created to improve the effectiveness of interventions aiming at increasing physical activity and healthy eating [11]. It was found in 2 systematic reviews using the taxonomy that interventions where participants engaged in self-monitoring were more effective in achieving goals of behavior change [11].

Potential harms of text messaging are generally limited but could potentially depend on the context and frequency of text messages. These might include, for example, perceptions of privacy invasion, and emotional trauma as a result of negative body image. This can be addressed by providing participant information before consent and access to services and resources designed to assist individuals in these issues.

### Serial Body Measurements in Weight Loss

Some reviews found that regular self-weighing was associated with weight loss. Despite variations in the frequency and size of correlation, the association with weight loss was consistent [12-14]. It has been suggested that the frequency for self-weighing to achieve successful outcomes is weekly [15]. However, current evidence does not conclude what the ideal frequency for self-weighing is despite most studies evaluating daily or weekly self-weighing [7]. There are other body measurements that can be taken serially in weight loss programs including BMI, waist and hip measurements, and WHRs. However, there is a significant gap in the literature regarding the effectiveness of these other measurements. Few studies conducted serial anthropometric measurements, and although text messaging was identified as an effective intervention [6,8,16], few combined it with anthropometric measurements. Research has shown the importance of WHRs in relation to obesity and health risks. For example, 1 study demonstrated that WHR is significantly associated with the risk of incident cardiovascular disease events and is a simple measure of abdominal obesity [17]. Similarly, another study found that WHR is associated with a higher risk of major adverse cardiovascular events among females, but not in males, with established coronary artery disease [18]. To our knowledge, there are no studies that have used WHR as a primary outcome measure in motivational text messaging studies. This study will therefore use WHR as an outcome measure.

The primary aim of this pilot study was to assess whether an 8-week program, consisting of weekly serial self-measurements of waist and hip circumference, combined with motivational text messages, could reduce WHR among Australian workers. Secondary aims were to examine the effects of the program on weight loss, exercise, eating behavior, and work-related well-being measures.

## Methods

### Trial Design

This pilot study evaluated the impact of an 8-week program consisting of motivational text messages and serial anthropomorphic measurements on reducing the WHR, other anthropometric measurements, health behaviors, and occupational health-related outcomes. It was a community-based, participant-blinded, staggered entry, parallel group study with balanced randomization (1:1) conducted in Australia using convenience sampling.

### Participants and Recruitment

Eligibility criteria were being above 18 years or older, being employed, and having access to a mobile phone in Australia. Exclusion criteria were people receiving weight-altering medications or participating in other weight loss programs. Participants were provided with a participant information sheet providing them with the length of the study, purpose, and affiliations of the study before enrollment into the study. Ethics approval was received from the Western Sydney Human Research Ethics Committee H11327.

Study recruitment ran from October 2016 to January 2017 via a Facebook page, emails to the researchers’ contacts, flyers to public notice boards and local businesses in the Northern Rivers, New South Wales, and information in councils’ newsletters in the Northern Rivers and Western Sydney region. Flyers and emails contained a link to an open a Web-based survey for participant enrollment and baseline data collection. The initial contact with the potential participants was thus made via the internet. Institutional affiliation to Western Sydney University was indicated in our materials. The recruitment materials advertised the study as a weight loss program, but there were no incentives offered to participants and participation was voluntary.

The survey was pretested on 18 volunteers to assess usability and technical functionality. Each participant completed identical baseline surveys, which consisted of 29 items over 7 pages. Items were not randomized or alternated. Adaptive questioning was not used. Only submitted surveys were considered as participant consent to the study. Respondents were able to review and change their answers while completing the survey but not after submission. No participant submitted more than 1 survey. No identifying information was linked to the data. ID numbers were used to analyze the data on password-protected computers.

### Intervention Group

Participants were randomly assigned to receive intervention or control messages for 8 weeks using a Web-based short message service SMS company. The intervention was a composite of motivational and self-monitoring messages. The 25 motivational messages sent every second day were based on promotion messages from another text-based study regarding nutrition [16], exercise, and monitoring (Multimedia Appendix 1). The self-monitoring messages were identical weekly requests for waist and hip circumference, aimed at providing self-feedback on progress (Multimedia Appendix 1) [15]. All messages were sent at 12 pm.

Participants were able to opt out of the intervention anytime by texting *STOP*.

### Control Group

Fortnightly control messages were sent with health information from the national guidelines on physical activity, diet, and nutrition [19]. Control group participants did not require to report their anthropometric measurements weekly. They only were requested to provide their WHR measurements at baseline and 8­week follow-up.

### Outcomes

Participants were followed up with a final Web-based survey 8 weeks after the start of their intervention. Final surveys were identical for all participants except for those completing it after Jan 23, 2017, when 2 open-ended questions were added to gather a more in-depth understanding about the pilot study. The final survey consisted of 23 items over 5 pages. Reminder emails and text messages were sent a week and a fortnight after completion. Two sets of participants were asked to complete the final survey outside of this protocol as a result of researcher error. This affected 10 participants; 6 participants were able to complete the survey 3 weeks after program completion instead of 2 weeks, whereas 4 participants were invited to complete the final survey 2 weeks before the completion of their intervention.

Outcome data was self-assessed and collected on the Web using SurveyMonkey at the beginning and end of the study. Questions were mainly derived from existing scales. The invitation to the final survey was sent in the last text message and email.

The primary outcome was WHR change from baseline to 8 weeks collected by participants measuring their waist and hip circumference in centimeters with help from instructive pictures and videos. Secondary outcomes were changes in anthropometric measurements, health behaviors, and occupational health-related outcomes. Self-reported health was measured with the global health question from the Short Form-36 (*In general, would you say your health is;* rated on a 5-point Likert scale ranging from *poor* to *excellent*). This item has consistently been found to possess strong psychometric properties compared with validated multi-item measures [20]. Parts of the widely used and validated Work Ability Index were used to measure occupational health [21]. Specifically, the 3-item version of the Work Ability Index, extensively validated by Mykletun and Furunes [22] was used and asked participants to self-report their current work ability; (1) on a scale from 0-10 compared with lifetime best, (2) in relation to physical demands, and (3) in relation to mental demands. Work ability at its lifetime best was measured through asking: *Work ability is an indication of how well your health, skills and experience match your current job demands. Assume that your work ability at its lifetime best has a value of 10 points. How many points would you give your current work ability? (0 means that you currently cannot work at all)*. The physical demands of work ability were measured by asking: *How do you rate your current work ability with respect to the physical demands of your work.* Answer categories ranged from *very good, rather good, moderate, rather poor, and very poor.* The same question was asked for the mental demands of the job*.* The commonly used and validated 9-item Emotional Exhaustion subscale from the Maslach Burnout Inventory (Human Services Survey) was used to keep the survey short and this often being regarded to be the core component of the Maslach Burnout Inventory [23]. The reliability coefficients for emotional exhaustion were 0.89 (frequency) and 0.86 (intensity) [24]. The Single Item Burnout scale was measured through the question*: On a scale from 0 to 10, how would you rate your current level of burnout?* where 1 represents *Not at all burnt out* and 10 represents *Extremely burnt out.* A previous study has demonstrated the validity of the Single Item Burnout scale. The item was highly and positively correlated with MBI-EE scores (r=0.8, *P*<.001) and was significantly associated with various outcome measures [25]. Productivity was measured through a self-developed 1-item question asking: *On a scale of 1-10, could you rate, how productive you were at work in the last week?* where 1 represents *Not at all productive* and 10 represents *Extremely productive.* Questions from a large cohort study, the 45 and Up Study, were used to measure healthy eating behavior, exercise, and total sitting hours per day [26].

Process measures were also assessed to measure levels of engagement and intervention uptake. These were as follows:

- The number of replies the intervention group made to the weekly request messages for self-measurement
- The time between finishing the study and completing the final survey

In addition, to elicit feedback on the program, the following 2 open-ended questions were added:

1. Do you feel that taking part in the study made you live or feel healthier? Can you explain?
2. Do you have any further comments on the program?

### Sample Size

To detect a difference in our participants’ WHR of 0.03 with a 5% significance level and assuming an SD of 0.064, 72 participants per group were required to provide the study with a power of 80%. However, our study included 30 participants at baseline per group because of unexpected difficulties in recruitment.

### Randomization

Participants were randomized in blocks of 10 to intervention or control through a computer-generated random number list on Excel created by a researcher (SWP) not involved in allocation. All other researchers were involved in allocation. Although there was no allocation concealment, enrollment of the participants occurred automatically during the baseline survey with no direct contact from the researchers.

Participants were allocated an ID number based on the order in which they completed the baseline survey. They were allocated to control or intervention on the Sunday after enrollment and started the intervention on the Monday in either the intervention or control group.

### Blinding

Data analysts and researchers undertaking randomization were not blinded during the trial; however, there was no direct contact between participants and researchers throughout the entirety of the trial, and ID numbers were used for participant anonymity during analysis. Participants were blinded to group allocation by concealing the frequency and content of text messages.

### Statistical Analysis

Baseline descriptive analyses examined variable distribution for sample characteristics. Continuous variables were presented as mean and SDs, and nonparametric data as median and interquartile ranges. Binomial and categorical variables were reported as proportions. Statistical analyses were performed on SPSS Version 22.0 (SPSS Inc, Chicago, IL, USA) and SAS version 9.4 (SAS Institute, Cary, NC, USA). *P* values less than .05 were considered significant. A mixed-model repeated measures analysis was performed to compare the effect of the intervention on the primary outcome measure, WHR, and other continuous secondary outcome measures at 8-week follow-up, compared with control. The interaction effect between *group* and *time* indicates whether the intervention successfully reduced WHR overtime compared with the control group. Compound symmetry was the specified covariance structure. This means that all the variances and covariances are equal for all participants. Healthy eating behaviors, exercise, and sitting hours per day were skewed to the right. These variables were analyzed to detect the presence of a negative or positive change from baseline to 8-week follow-up. The intervention effect was then compared by using the Fisher exact test because of the small numbers in each category. Atypical data such as impossible body measurement values were considered as missing values, and these numbers were excluded from analysis.

## Results

### Recruitment

**Figure 1:**
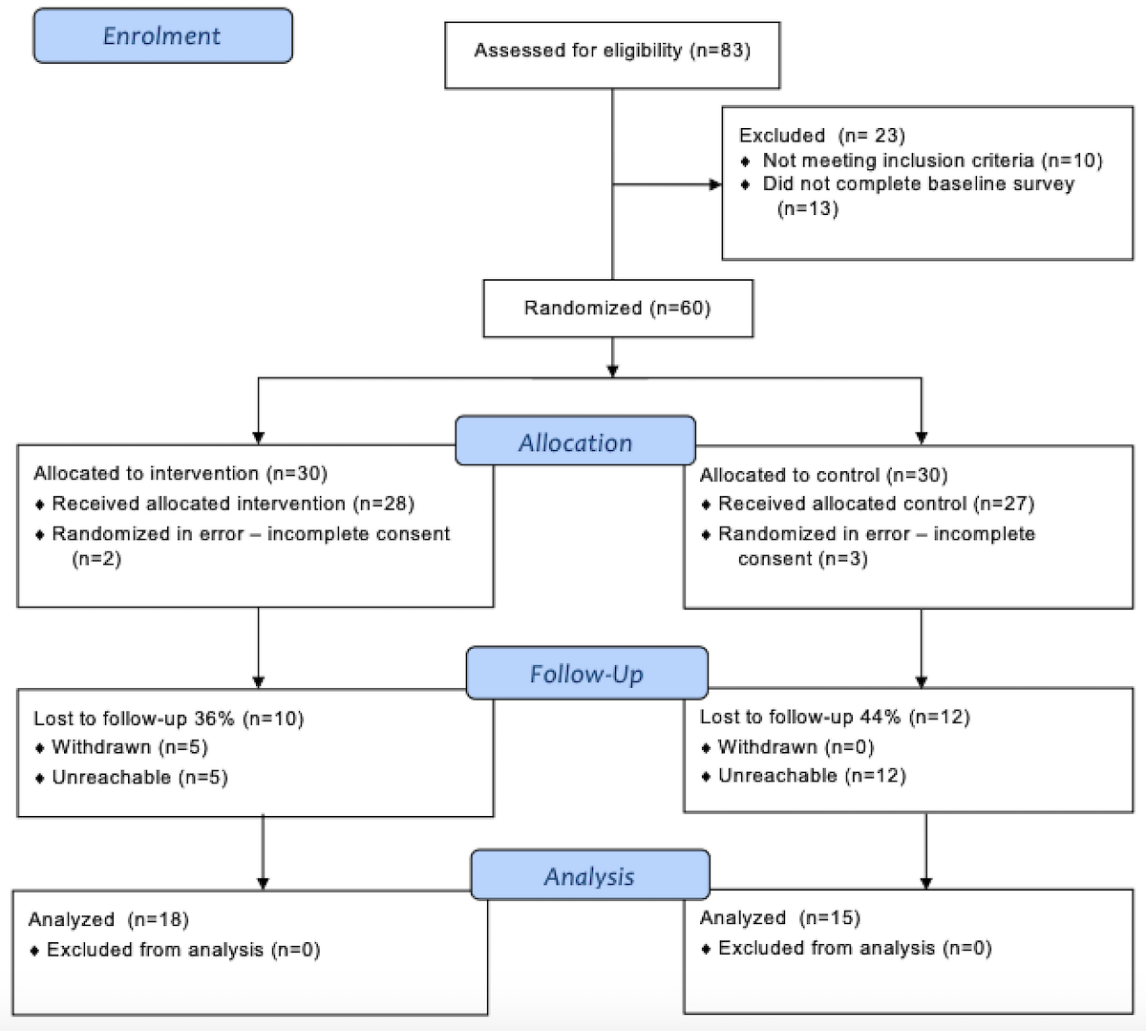
Participant flowchart.

This study recruited members between October 2016 and January 2017. There were 9 weekly sets of participants who entered the trial throughout this recruitment period, with the final set of participants completing the intervention in March 2017. Participant numbers each week varied from 1 to 18. The trial was ended as per the scheduled date of closure. The participant flow is summarized in Figure 1.

### Baseline

A total of 55 participants entered the trial, 33 completed the trial and were included in the final analyses. Baseline characteristics appear to vary between the intervention and control group (Tables 1 and 2). The intervention group was older (median age 45 vs 33 years) and overall less healthy than the control group, with higher mean hip circumference (106.1 vs 95.7 cm), mean weight (76.9 vs 69.9 kg), and proportion of overweight and obese BMI 52% (11/21) versus 39% (9/23); and lower moderate physical activity levels (median: 30 vs 60 min/week).

### Numbers Analyzed

Of the 60 participants randomized, 5 participants were randomized in error (3 intervention and 2 control group participants), 5 participants withdrew during the program and 22 participants were lost to follow-up (ee Figure 1). Of these participants lost to follow-up, 5 provided partially completed final surveys at 8 weeks. Data from partially completed surveys were used in the analysis where possible. These participants completed the first page of the final survey only. All 5 participants did not enter weight and waist or hip circumference measurements. Therefore, only the number of measurements for sitting time in the analyses was higher than the other variables. Completed final surveys were provided by 33 participants and used in primary outcome analysis.

**Table 1:** Baseline participant characteristics.

Characteristics		Intervention (n=23)	Control (n=27)	Total (n=50)
**Sociodemographics**				
	Age (years), median (Q1, Q3)	45 (30, 53)	33 (25, 46)	38 (27,51)
	Female, n (%)	19 (83)	23 (85)	42 (84)
	Hours spent doing paid work, median (Q1, Q3)	32 (20, 40)	35 (30, 40)	33.5 (24, 40)
**Relationship status, n (%)**				
	In a relationship or married or engaged or de facto^a^	11 (50)	14 (52)	25 (51)
	Single or divorced or widowed or separated^a^	11 (50)	13 (48)	24 (49)
**Anthropometric measures**				
	Height, cm^b^, mean (SD)	164.9 (8.3)	166.0 (9.2)	165.5 (8.7)
	Weight, kg^b^, mean (SD)	76.9 (20.4)	69.9 (21.4)	73.2 (21.0)
	Body mass index^b^, kg/m^2^, mean (SD)	28.2 (6.7)	25.1 (6.1)	26.6 (6.5)
	Overweight status^b^, n (%)	11 (52)	9 (39)	20 (45.5)
	Waist circumference, cm^c^, mean (SD)	89.4 (16.1)	80.4 (17.2)	85 (17.0)
	Hip circumference, cm^c^, mean (SD)	106.1 (13.4)	95.7 (13.5)	101.0 (14.3)
	Waist-to-hip ratio^c^, mean (SD)	0.84 (0.08)	0.84 (0.10)	0.83 (0.09)
**Health behaviors**				
	Number of serves of cooked vegetables, median (Q1, Q3)	2 (1, 3)	1 (1, 2)	2 (1, 3)
	Number of serves of raw vegetables, median (Q1, Q3)	1 (1, 1)	2 (1, 2)	1 (1, 2)
	Met vegetables requirement [27], n (%)	4 (18)	5 (19)	9 (18)
	Number of serves of fruit, median (Q1, Q3)	2 (1, 2)	1 (1, 2)	1 (1, 2)
	Number of glasses of fruit juice^d^, median (Q1, Q3)	0 (0, 0)	0 (0, 0)	0 (0, 0)
	Met fruit requirement [27], n (%)	12 (55)	13 (48)	25 (51)
	Hours spent sitting per day, mean (SD)^e^	8 (7, 10)	8 (6, 12)	8 (7, 10)
	Mild physical activity per week (min)^f^, median (Q1, Q3)	60 (14, 240)	60 (10, 120)	60 (10, 180)
	Moderate physical activity per/week (min)^g^, median (Q1, Q3)	60 (10, 180)	30 (20, 60)	60 (10, 120)
	Vigorous physical activity per/week (min)^h^, median (Q1, Q3)	2 (0, 60)	20 (0, 60)	7 (0, 60)
	Proportion meeting physical activity guidelines [27], n (%)	12 (55)	14 (52)	26 (53)
	Accountability partner, n (%)	6 (27)	7 (26)	13 (27)

^a^n=49 (control: 27 vs intervention: 22)*.*

^b^n=44 (intervention: 21 vs control: 23).

^c^n=39 (intervention: 20 vs control: 19).

^d^n=47 (intervention: 20 vs control: 27).

^e^n=50.

^f^n=47 (intervention: 21 vs control: 26).

^g^n=46 (intervention: 21 vs control: 25).

^h^n=48 (intervention: 21 vs control: 27).

**Table 2:** Baseline occupational health-related outcomes.

Outcomes		Intervention (n=23)	Control (n=27)	Total (n=50)
General health, n (%)^a^				
	Excellent or very good	6 (27)	8 (31)	14 (29)
	Good	10 (46)	13 (50)	23 (48)
	Fair or poor	6 (27)	5 (19)	11 (23)
Work ability lifetime best (0 to 10), median (Q1, Q3)		8 (7, 9)	8 (7, 9)	8 (7, 9)
Work ability mental demands, n (%)				
	Very good	3 (14)	7 (26)	10 (20)
	Rather good	12 (55)	14 (52)	26 (53)
	Moderate or rather poor or very poor	7 (32)	6 (22)	13 (27)
Work ability physical demands, n (%)				
	Very good, n (%)	9 (41)	14 (52)	23 (47)
	Rather good, n (%)	10 (46)	10 (37)	20 (41)
	Moderate or rather poor or very poor, n (%)	3 (14)	3 (11)	6 (12)
Productivity (0 to 10), median (Q1, Q3)		7 (6, 8)	8 (6, 9)	7 (6, 8)
Burnout Score (0 to 10), mean (SD)		4.8 (2.6)	4.7 (2.6)	4.8 (2. 7)
Maslach burnout inventory—emotional exhaustion, mean (SD)		31.0 (13.6)	30.4 (11.5)	30.7(12.4)

^a^n=48 (intervention: 22 vs control: 26).

**Table 3:** Comparison between control group and intervention group anthropometric measures and work-related health.

Outcomes			Intervention		Control		Time effect	Group × time effect	Group effect
			Baseline, mean (SE)	At 8 weeks, mean (SE)	Baseline, mean (SE)	At 8 weeks, mean (SE)	*P* value	*P* value	*P* value
**Primary outcome**									
	Waist-to-hip ratio		0.84 (0.02)	0.86(0.02)	0.83(0.02)	0.82(0.03)	.68	.30	.43
	**Anthropometric measures**								
		Body mass index (kg/m^2^)	28.25 (1.34)	28.14 (1.34)	25.11 (1.29)	25.26 (1.29)	.85	.22	.12
		Waist circumference (cm)	89.95 (3.73)	90.28 (3.83)	80.76 (3.88)	77.00 (4.26)	.45	.36	.04
		Hip circumference (cm)	107.08 (3.24)	104.12 (3.34)	96.39 (3.36)	93.31 (3.76)	.17	.97	.02
**Well-being and work-related health**									
	Burnout score (0 to 10)		4.81 (0.57)	3.11 (0.63)	4.77 (0.52)	3.60 (0.67)	.004	.57	.75
	Maslach burnout inventory—emotional exhaustion		25.50 (2.72)	30.82 (2.74)	30.37 (2.37)	31.00 (2.62)	.08	.11	.39
	Work ability (0 to 10)		7.91 (0.36)	7.65 (0.45)	8.11 (0.32)	8.31 (0.41)	.91	.41	.35
	Productivity (0 to 10)		7.18 (0.39)	6.65 (0.45)	7.25 (0.35)	7.92 (0.47)	.86	.15	.14

### Post Results

The changes in anthropometric measures and work-related health are summarized in Table 3. The group by time effect was not significant indicating the pattern over time did not differ by group for the primary outcome (Table 3, group by time). The main effect of time was also not significant for all variables, indicating no change over time for either group, except for the single item burnout where there was a significant decrease in burnout over time (mean [SE]: pre 4.80 [0.39] vs post 3.36 [0.46]; *P*=.004). Only waist and hip circumferences had a significant group effect, indicating that these measurements averaged over time were significantly greater in the intervention group.

**Table 4:** Comparison between control group and intervention group for health behaviors, representing the number (%) of participants that reported changes in health behaviors from baseline to 8-week follow-up.

Health behaviors		Less, n (%)	Same, n (%)	More, n (%)	*P* value
**Number of serves of cooked vegetables^a^**					
	Intervention	4 (24)	10 (59)	3 (18)	.26
	Control	2 (13)	6 (40)	7 (47)	—^a^
**Number of serves of raw vegetables^a^**					
	Intervention	0 (0)	7 (39)	11 (61)	.08
	Control	3 (20)	7 (47)	5 (33)	—
**Number of serves of fruit**					
	Intervention	2 (11)	10 (56)	6 (33)	.41
	Control	0 (0)	7 (47)	8 (53)	—
**Number of glasses of fruit juice**					
	Intervention	1 (6)	14 (88)	1 (6)	.79
	Control	1 (7)	12 (80)	2 (13)	—
**Total minutes of moderate or vigorous exercise**					
	Intervention	6 (33)	3 (17)	9 (50)	.38
	Control	6 (40)	0 (0)	9 (60)	—
**Total hours sitting per day**					
	Intervention	6 (33)	3 (17)	9 (50)	.63
	Control	7 (40)	2 (0)	8 (60)	—

^a^Not applicable.

No statistically significant differences were detected for health behaviors between the 2 groups (Table 4). However, it is interesting to note there was an increase, from baseline to 8-week follow-up, in the number of serves of raw vegetables consumed by 11 out of 18 participants (11/18, 61%) in the intervention group with no decrease of serves, whereas 5 out of 15 participants (5/15, 33%) in the control group increased their servings of raw vegetables and 3 out of 15 (3/15, 20%) decreased. No participant had significantly poorer health behaviors at the end of the study or other harms found as an outcome of this study.

### Process Measures

The pattern of intervention uptake is shown in Table 1 in Multimedia Appendix 2. Intervention group participants’ responses to weekly self-measurements requests followed an overall downward trend with the peak percentage of 61% (14/23) in week 3 and the lowest percentage of 35% (8/23) in week 8. It was also found that participants in the control group took longer to complete the final survey compared with the intervention group after the 8 weeks with a mean of 8.80 days compared with 3.17 days (Table 2, Multimedia Appendix 2).

### Participant Feedback

A total of 15 people responded to the qualitative question (intervention=8 vs control=7).

#### Overall Findings

A total of 11 participants felt that the study did not make them live or feel healthier and had *no impact*. Feedback included:

... *text came through whilst at work so didn’t influence exercise pattern, also over Xmas period so exercised less and ate more* and *I think more frequent texts, even daily, with a list of exercises for the day or positive affirmations for regular exercising and why you should do the days 30 min exercise plan (for example)*.

#### Intervention Group

In total, 5 of 8 participants who responded commented that the study did not make them live or feel healthier. They commented:

Unfortunately no. Although I found the texts very informative, I didn’t make an effort to put them into practice

No, not really. The texts were too easy for me to ignore, or forget about. I’m not in the habit of checking my phone regularly

In total, 3 participants thought the intervention had a positive impact, for example:

Yes as I thought about my health more often

It has been positive to have daily texts and reminders even if I took little action from them

#### Control Group

In total, 6 of 7 responses were negative. Some felt that:

...it would have been better to have more frequent texts

I don’t know think one quick fact every couple of weeks can change a whole attitude. For me, it requires more regular reminders and having someone like an accountability partner who can follow you up often works best

In total, 1 had a positive response to the study:

yes it was a good reminder to eat healthy and exercise

## Discussion

### Principal Findings

No significant changes over time were found between the WHR of the intervention and control group over time. Single item burnout showed a significant decrease over time. No weight gain or other anthropometric measurements, health behaviors, and occupational health measures showed significant changes over time.

A number of mechanisms might account for the results of this study. First, the study ran over the holiday season in Australia, which is traditionally a time in which individuals gain weight [28]. One study on diabetes prevention advised dietitians that the goal for patients in this period should be weight stabilization [29]. No weight gain or WHR change was found at the 8-week follow-up, suggesting that weight stabilization might have occurred.

The decrease in burnout independent of allocation might similarly be a reflection of upcoming major holidays. There is little evidence to suggest that upcoming holidays decrease burnout; however, a study found that if an individual had a trip planned for their holiday time, they were more likely to report being happier [30].

The diversity of interventions makes direct numerical comparisons difficult in technological weight loss studies. However, text messaging use for reminders, such as those we used to induce self-monitoring or to promote behavior change, does have an evidence basis. One systematic review found that although a relatively new area of research, text messaging as a lifestyle intervention was promising in its feasibility and acceptability [9]. However, of the 10 studies reviewed measuring weight or BMI as an outcome, only 5 showed a statistical and clinical difference after the intervention. The sole study in the review that used WHR found no difference when using text messages to remind participants of physical exercise goals they had set [31]. In reviewing the effect of text messaging on physical activity, the systematic review found that 3 of the 6 trials that used physical activity as an outcome showed a statistically significant increase in the frequency or duration [9]. With regard to diet, 3 out of the 4 studies with dietary outcomes showed a statistically significant improvement using text messaging. More recently, a pilot study examining the use of text messaging to improve health among African American women, an at-risk group for obesity, found it to be effective [32]. Self-monitoring as an intervention has been shown to be effective with 1 study finding that self-weighing was associated with weight loss [12], a result we did not establish with self-measuring WHR.

Burnout reduction using text messages among workers has, to the researchers’ knowledge, never been investigated before in a randomized controlled trial (RCT) setting. This suggests that this maybe an area of further research to further explore its impact given that it significantly reduced overtime in both groups. This might have been a type 2 error though. Other intervention studies have tested the efficacy of guided Web-based and mobile-based stress management training for employees and found that emotional exhaustion was reduced in the intervention group [33]. Similarly, a 2018 RCT evaluated the efficacy of an internet-based, app-supported stress management intervention for college students and also found a reduction in emotional exhaustion among intervention group participants [34]. Along with this, most studies excluded participants with normal BMIs. One trial that included these participants, similar to our study, found that though text messages increased physical exercise significantly, there was no impact on BMI [35]. In addition, another study found that their intervention had significantly less effect on those with lower BMIs when compared with those in the obese range [36]. Finally, a 2017 study among obese adolescents that used a mobile phone–based intervention consisting of 3 parts—use of the Fitbit Flex, delivery of an online educational program, and biweekly text messages during the maintenance phase—also found that the program significantly improved BMI, physical activity days per week, and servings of fruits and vegetables per day [37]. This could offer further explanation for our nonsignificant findings.

### Limitations

This study had several limitations which must be considered when reviewing the results and in the development of future research. The restricted recruitment time limited the number of participants and contributed to a low power. Participant enrollment in the study was also limited by access to tools such as a tape measure. However, we compensated for this by the provision of videos explaining how to use string and a ruler to measure WHR. Another limitation of this study was the differing baseline characteristics between groups. The intervention group had a higher proportion of overweight and obese BMIs than the control group (52% vs 39%), a higher weight (76.9 kg vs 69.9 kg), and a wider hip circumference (106.1 cm vs 95.7 cm) in baseline characteristics. Furthermore, there was a significant difference between the waist and hip measurements averaged over time for the 2 groups, with the intervention group having significantly greater waist and hip measurements (*P*=.04 and *P*=.02, respectively).

Although our lost to follow-up rates were relatively high 40% (22/55), a review focusing on Web-based weight loss interventions showed most had an attrition rate that was higher [38]. Another study showed that 55% was a usual rate in obesity trials [39], which brings our lost to follow-up into perspective**.** However, there was a difference in the rates of participants who completed the intervention but not the final survey. There was a lower proportion in the intervention group (n=5 [18%]) than control (n=12 [44%]). These differing unreachable rates could be because of the different frequencies of messages between the groups and indicates that fortnightly contact was not enough to maintain engagement in the control group. This was confirmed in the feedback of the control group forgetting they were in the study. It is interesting to note, however, that all the participants who withdrew from the study were in the intervention group, despite their high levels of contact. This might be due to the fact that some participants found the frequency of text messages intrusive, though this was not reflected in the feedback.

Another reason for the unequal attrition rates was suggested by 1 study that examined the reasons behind dropout [40]. They found that lower initial weight loss was associated with attrition [40]. Therefore, the lost to follow-up rates might have been higher in the control group because of the poor efficacy of the control text messages and lack of weight loss. This might provide an explanation for the results, as those in the control group who achieved weight reduction were more likely to complete the final survey and be included in the analysis.

All partial completers of the final survey (n=5) dropped out when asked to provide their weight and waist and hip circumference. It might be useful for future studies to consider placing questions known to lead to low response rates at the end of the survey. However, in our case, it was the primary outcome measure, so in future trials other methods of collecting weight and WHR data might need to be considered to ensure valid and complete data are collected.

Process measures to test engagement in the study were used to assist with the development of further research into this area. Follow-up response times were a part of these, and we found the intervention group had a shorter response time than the control group (mean days [SD]: 3.17 [3.50] vs 8.80 [6.27]). This presents an important aspect to consider when running similar trials in the future as it suggests that altering the content rather than the frequency of the messages between the groups might be a more effective option.

The other process measure of this study was the replies from the intervention group to the request messages. Serial self-measurement was a key intervention in this study, and strong uptake would be needed to measure its efficacy. However, replies to these messages were shown to decrease over time, revealing a decreasing level of engagement. Reasons for this could be similar to reasons for lost to follow-up events as discussed previously. This decreasing intervention uptake might have contributed to our negative findings and should be considered when interpreting results.

Some aspects of this study limit its application to the wider Australian context. First, 84% of the population was female, which, although a common problem with many weight loss studies [41], restricts generalizability if the target population is dissimilar. However, 1 systematic review found that lifestyle interventions, like ours, are effective in both men and women [42].

This study also had a limited, primarily young, age range. This might be because of the nature of our recruitment via social media and by restricting this study population to workers. Younger participants might also feel more comfortable participating in a study involving a relatively new aspect of technology. Another study limitation is self­reported measurements. We included several strategies for accuracy for the primary outcome measure. First, before starting the survey, we advised people we would ask them to measure their waist and hip and asked them to be in a comfortable place to measure themselves. Second, we showed them a picture and a video during the Web-based survey on how to measure hip and waist circumference to assist people in completing their measurements. Nonetheless, it is likely that some people will have estimated their hip and waist circumference, which might have biased the results. Secondary outcome measures were mainly based on validated scales for work ability [21], emotional exhaustion [24], single item burnout [25], or questions derived from a large cohort study for sitting behavior, healthy eating, and exercise [26].

### Conclusions

This study is an innovative pilot trial using text messaging and serial self-measurement in weight management. The results did not detect a change in WHR ratio in Australian workers over 8 weeks. However, these results should be interpreted in the context of limited sample size and decreasing intervention uptake over the course of the study. We are unable to conclude this intervention is not effective. A larger sample would be necessary to see if the combination of these interventions is effective. The findings around study design and participant interaction with the interventions are useful for informing and contributing to the design of future studies and the growing body of literature on serial self-measurements combined with text messaging.

## Appendix

Multimedia Appendix 1 Text messages.

Multimedia Appendix 2 Process measures.

Multimedia Appendix 3 CONSORT-EHEALTH checklist (V 1.6.1).

## Abbreviations

BMI: body mass index
RCT: randomized controlled trial
WHR: waist-to-hip ratio
