# Supplementary material for: Effect of Serial Anthropometric Measurements and Motivational Text Messages on Weight Reduction Among Workers: Pilot Randomized Controlled Trial
Source: JMIR Mhealth Uhealth. 2019 Apr 24;7(4):e11832. doi: 10.2196/11832 (PMC6505373; doi:10.2196/11832)
Supplement: Multimedia Appendix 1 [file mhealth_v7i4e11832_app1.docx]

*Source:* [16], [17].

|  | **Intervention** | **Control** |  | **Intervention (request measurement)** |
| --- | --- | --- | --- | --- |
| **Day 1: Monday following submission of entry survey** | Welcome to Text4Health! You will receive texts & asked weekly to measure your hip & waist. If you didn’t request this/wish to withdraw at any time, text STOP. | Welcome to Text4Health! You will receive your first health message soon. If you didn’t request this/wish to withdraw at any time, text STOP. | **Day 1: Monday following submission of entry survey** |  |
| **Day2** |  |  | **Day2** |  |
| **Day3** | Weigh yourself daily at the same time and on the same scale. This is important. Checking on your progress will help you control your weight. |  | **Day3** |  |
| **Day4** |  |  | **Day4** |  |
| **Day5** | Small diet changes add up. Eat breakfast every day. You will eat less during the day and it will help you reach your weight loss goals. |  | **Day5** |  |
| **Day6** |  |  | **Day6** |  |
| **Day7** | Stay healthy. Use the “plate method”. Fill up ½ your plate with vegetables, ¼ with starch, ¼ with protein. |  | **Day7** | Please send your waist and hip measurements in CENTIMETERS at your earliest convenience. Please follow this example: Waist 100 Hip 130 |
| **Day8** |  |  | **Day8** |  |
| **Day9** | Check your food intake. Record everything you eat and portion sizes. Checking increases awareness of what you are eating. |  | **Day9** |  |
| **Day10** |  |  | **Day10** |  |
| **Day11** | Count steps to increase the amount you walk. Use a pedometer. Set a goal of adding 150 steps a day up to 5000 steps a day or 4kms. |  | **Day11** |  |
| **Day12** |  |  | **Day12** |  |
| **Day13** | Use smaller plates. You will still clean your plate, feel satisfied, and have better portion control. |  | **Day13** |  |
| **Day14** |  | Thank you for participating in Text 4 Health! Regular exercise can reduce health problems such as heart disease.We will contact you again in 14 days. | **Day14** | Please send your waist and hip measurements in CENTIMETERS at your earliest convenience. Please follow this example: Waist 100 Hip 130 |
| **Day15** | Keep track of the things that lead to unplanned and overeating. |  | **Day15** |  |
| **Day16** |  |  | **Day16** |  |
| **Day17** | Activity burns calories and helps maintain weight. Climbing stairs, parking further away, or walking to the office add up quickly to 30 mins a day. |  | **Day17** |  |
| **Day18** |  |  | **Day18** |  |
| **Day19** | Eat slowly. Put fork down between bites. Check fullness level during meal. When full push your plate away. Satisfaction takes 15-20 min. |  | **Day19** |  |
| **Day20** |  |  | **Day20** |  |
| **Day21** | Keep exercising! After 1 year, dieters who exercise maintain most of their original weight loss. |  | **Day21** | Please send your waist and hip measurements in CENTIMETERS at your earliest convenience. Please follow this example: Waist 100 Hip 130 |
| **Day22** |  |  | **Day22** |  |
| **Day23** | Select healthy breakfast cereals. Follow the “5 and 5” rule—5 grams fibre & 5 or less of sugar. Or try heart-healthy oatmeal with honey! |  | **Day23** |  |
| **Day24** |  |  | **Day24** |  |
| **Day25** | Monitor your progress and how you are doing. Schedule time to review your progress in your calendar. |  | **Day25** |  |
| **Day26** |  |  | **Day26** |  |
| **Day27** | Exercise helps more than just with weight loss. It helps to decrease high blood pressure, improve diabetes, and decrease cholesterol. |  | **Day27** |  |
| **Day28** |  | Thank you for participating in Text 4 Health! Do 2.5 -5hrs of moderate exercise wkly. We will contact you again in 14 days. | **Day28** | Please send your waist and hip measurements in CENTIMETERS at your earliest convenience. Please follow this example: Waist 100 Hip 130 |
| **Day29** | Reward yourself. It’s OK to have a little sweet foods such as cake, biscuits, and lollies, and alcohol. |  | **Day29** |  |
| **Day30** |  |  | **Day30** |  |
| **Day31** | Cross train. Vary your exercise: walk, bike, elliptical, water /chair aerobics. It will help you stay motivated and have fun! |  | **Day31** |  |
| **Day32** |  |  | **Day32** |  |
| **Day33** | Identify and change habits and foods that lead to binges including risky foods kept in the house such as chips or watching TV while eating. |  | **Day33** |  |
| **Day34** |  |  | **Day34** |  |
| **Day35** | Stay on top of how you are doing. Review your progress to check for patterns. Monitor at least 2-3 times a week. |  | **Day35** | Please send your waist and hip measurements in CENTIMETERS at your earliest convenience. Please follow this example: Waist 100 Hip 130 |
| **Day36** |  |  | **Day36** |  |
| **Day37** | For general fitness: exercise 30 minutes daily. 8-10 exercises, 8-15 repetitions, 1-3 sets, 30-90 second rest between sets. |  | **Day37** |  |
| **Day38** |  |  | **Day38** |  |
| **Day39** | Use a grocery list for grocery shopping and only buy planned for items. This will help you buy good healthy foods. |  | **Day39** |  |
| **Day40** |  |  | **Day40** |  |
| **Day41** | Limit size of portions at mealtimes by measuring planned servings. Keep measuring utensils readily available. |  | **Day41** |  |
| **Day42** |  | Thank you for participating in Text 4 Health! Do muscle strengthening activities on at least 2 days each week. We will contact you again in 14 days. | **Day42** | Please send your waist and hip measurements in CENTIMETERS at your earliest convenience. Please follow this example: Waist 100 Hip 130 |
| **Day43** | Weigh yourself daily. Place the weight on a graph to see trends over time. It is natural to fluctuate daily due to things such as water. |  | **Day43** |  |
| **Day44** |  |  | **Day44** |  |
| **Day45** | Plan meals in advance to increase self-awareness—3 meals and up to 2 snacks per day going no longer than 4-5 hours between eating. |  | **Day45** |  |
| **Day46** |  |  | **Day46** |  |
| **Day47** | Any exercise is better than no exercise. Use a strategy to find a way to get in at least some exercise. |  | **Day47** |  |
| **Day48** |  |  | **Day48** |  |
| **Day49** | Stay motivated! At the beginning of the week plan your exercise sessions and treat them like you would any other appointment. |  | **Day49** | Please send your waist and hip measurements in CENTIMETERS at your earliest convenience. Please follow this example: Waist 100 Hip 130 |
| **Day50** |  |  | **Day50** |  |
| **Day51** | Use restaurant strategies: 2 vegetable servings, 1 caloric beverage. If calories are listed keep meals below 800. |  | **Day51** |  |
| **Day52** |  |  | **Day52** |  |
| **Day53** | When you don’t feel like working out, bargain with yourself to exercise for just 10 minutes then see how you feel. |  | **Day53** |  |
| **Day54** |  |  | **Day54** |  |
| **Day55** | Make small changes. Use trade-offs such as: I will have dessert every OTHER night or, I will only eat half of the dessert. |  | **Day55** |  |
| **Day56** | Thank you for participating in Text4Health, please click here https://www.surveymonkey.com/r/6WRWDGZ or the link in your email to complete the final survey. | Thank you for participating in Text4Health, please click here https://www.surveymonkey.com/r/6WRWDGZ or the link in your email to complete the final survey. | **Day56** | Please send your waist and hip measurements in CENTIMETERS at your earliest convenience. Please follow this example: Waist 100 Hip 130 |
| **Reminder** | Hi! We didn’t get your final survey; please click on the link to fill it out now. https://www.surveymonkey.com/r/6WRWDGZ | Hi! We didn’t get your final survey; please click on the link to fill it out now. https://www.surveymonkey.com/r/6WRWDGZ |  |  |
| **If Texted STOP** | Thank you, your participation in this study has been withdrawn. | Thank you, your participation in this study has been withdrawn. | **If Texted STOP** |  |
|  |  |  |  |  |
