# Supplementary material for: Effect of Serial Anthropometric Measurements and Motivational Text Messages on Weight Reduction Among Workers: Pilot Randomized Controlled Trial
Source: JMIR Mhealth Uhealth. 2019 Apr 24;7(4):e11832. doi: 10.2196/11832 (PMC6505373; doi:10.2196/11832)
Supplement: Multimedia Appendix 2 [file mhealth_v7i4e11832_app2.docx]

# Appendix 1: Process measures

Table 1. Process measure: intervention uptake

| Week | Participant replies (n (%)) |
| --- | --- |
| 1 | 11 (48) |
| 2 | 12 (52) |
| 3 | 14 (61) |
| 4 | 12 (52) |
| 5 | 10 (43) |
| 6 | 12 (52) |
| 7 | 9 (39) |
| 8 | 8 (35) |

Table 2. Process measure: participant follow-up times

|  | Intervention (n=18) | Control (n=15) | Total (n=33) |
| --- | --- | --- | --- |
| Time taken to complete follow-up survey  (mean days (SD)) | 3.17 (3.50) | 8.80 (6.27) | 5.73 (5.64) |
